# The *ERBB2* gene polymorphisms rs2643194, rs2934971, and rs1058808 are associated with increased risk of gastric cancer

**DOI:** 10.1590/1414-431X20198379

**Published:** 2019-05-16

**Authors:** K.C. Vázquez-Ibarra, A.R. Bustos-Carpinteyro, A. García-Ruvalcaba, M.T. Magaãa-Torres, R. Gutiérrez-Aguilar, M.E. Marín-Contreras, E. Santiago-Luna, J.Y. Sánchez-López

**Affiliations:** 1 Division of Genetics, Western Biomedical Research Center, Mexican Institute of Social Security, Guadalajara, Jalisco, Mexico; 2 Biology Division, University Center of Biological and Agricultural Sciences, University of Guadalajara, Zapopan, Jalisco, Mexico; 3 Doctoral Program in Human Genetics, Department of Molecular Biology and Genomics, University Center of Health Sciences, University of Guadalajara, Guadalajara, Jalisco, Mexico; 4 Department of Gastroenterology, National Medical Center of the West, Mexican Institute of Social Security, Guadalajara, Jalisco, Mexico

**Keywords:** *ERBB2* gene, Gastric cancer, HER2, Polymorphisms

## Abstract

Gastric cancer (GC) is the third most lethal type of cancer worldwide. Single nucleotide polymorphisms (SNPs) in regulatory sites or coding regions can modify the expression of genes involved in gastric carcinogenesis, as *ERBB2*, which encodes for the tyrosine-kinase receptor HER-2. The aim of this work was to analyze the association of the polymorphisms: rs2643194, rs2517951, rs2643195, rs2934971, and rs1058808 with GC, as they have not yet been analyzed in GC patients, as well as to report their frequency in the general Mexican population (GMP). We studied genomic DNA from subjects with GC (n=74), gastric inflammatory diseases (GID, n=76 control subjects), and GMP (n=102). Genotypes were obtained by means of real-time PCR and DNA-sequencing. The risks for GC were estimated through odds ratio (OR) using the Cochran-Armitage trend test and multinomial logistic regression. Increased risk for GC was observed under the dominant inheritance model for the rs2643194 TT or CT genotypes with an OR of 2.75 (95%CI 1.12−6.75, P=0.023); the rs2934971 TT or GT genotypes with an OR of 2.41 (95%CI 1.01−5.76, P=0.043), and the rs1058808 GG or CG genotypes with an OR of 2.21 (95%CI 1.00−4.87, P=0.046). The SNPs rs2643194, rs2934971, and rs1058808 of the *ERBB2* gene were associated with increased risk for GC.

## Introduction

Gastric cancer (GC) is the fifth most common type of cancer worldwide, and is the third most lethal type of cancer (1). Chronic gastritis, the inflammation of the mucosal layer of the stomach, is the first step of the multistep cascade of the onset of GC (non-atrophic chronic gastritis, multifocal atrophic gastritis, intestinal metaplasia, dysplasia, and cancer) related to *Helicobacter pylori* infection (2). Environmental and biological factors such as diet, smoking, toxic exposure, and *H. pylori* and Epstein-Barr virus infections are involved in all of these disease etiologies. Gastric carcinogenesis can be caused by the accumulation of genetic and epigenetic abnormalities, which modify the expression of different types of genes important for cell regulation, such as oncogenes and those encoding DNA repair molecules, tumor suppressor factors, and cell growth and cell adhesion molecules (3).

*ERBB2* is a gene frequently altered in different types of cancer. It is located at chromosomal region 17q12 and has 32 exons. It encodes for the human epidermal receptor-2 (HER-2), which consists of 1255 residues, has a molecular weight of 137.9 kDa, and belongs to a family of tyrosine kinase receptors. Structural and functional alterations of the HER-2 protein have been reported in different stages of carcinogenesis, such as initiation, promotion, and progression. HER-2 amplification and overexpression were reported for the first time in breast cancer and were significantly associated with poor prognosis. Additional studies have shown that HER-2 is also present in other malignancies, including colorectal, ovarian, prostate, and lung cancers, and, in particular, gastric cancer and gastroesophageal cancer (4).

Allelic variations as single nucleotide polymorphisms (SNP) in regulatory sites or coding regions could modify the HER-2 expression, affecting the progression of chronic gastritis and other premalignant lesions involved in the multistep cascade of gastric carcinogenesis. Several authors have analyzed the association of the *ERBB2* polymorphisms rs2643194 (c.−18+1614 C>T), rs2517951 (c.−18+1663 C>T), rs2643195 (c.−18+1684 A>G), rs2934971 (c.−18+3073 G>T), and rs1058808 (c.3418 C>G) with some types of cancer (5–11); however, these have not been analyzed in patients with GC. The aim of this study was to analyze the association of the *ERBB2* polymorphisms rs2643194, rs2517951, rs2643195, rs2934971, and rs1058808 with GC, and to report the gene frequency in the general Mexican population (GMP).

## Material and Methods

We studied three groups of subjects: 1) GC group: 74 patients with gastric adenocarcinoma (diffuse type n=35, intestinal type n=26, mixed type n=6, undetermined type n=4), whose diagnoses were made by a pathologist through analysis of biopsies of gastric tumor obtained by endoscopy. 2) GID (gastric inflammatory diseases) group: as controls, we studied 76 subjects with chronic gastritis, chronic atrophic gastritis, or intestinal metaplasia, in whom malignancy was discarded by a pathologist through analysis of gastric biopsies. Both GC and GID groups were recruited from the gastroenterology departments of four hospitals belonging to Instituto Mexicano del Seguro Social in the city of Guadalajara, Jalisco. 3) GMP group: 102 individuals, adults, healthy and unrelated that were recruited from the Centro Medico Nacional de Occidente, Instituto Mexicano del Seguro Social blood bank.

The study was approved by the local Committee of Health Research and Ethics of the Centro de Investigación Biomédica de Occidente, Instituto Mexicano del Seguro Social (CLIEIS-1305). All subjects provided written informed consent.

Genomic DNA was extracted following the salting-out method (12) from a sample of 5 mL of peripheral blood. Genotyping of the SNPs rs2643194, rs2517951, and rs2643195 was done by polymerase chain reaction (PCR) amplification with the forward (5′-AAGCATGGCGTCCACA-3′) and reverse (5′-CATCGGGATGTTAGGATCA-3′) primers. PCR reaction was performed in a 25-μL reaction mixture comprising 200 ng genomic DNA, 5 pM each primer, 0.5 U Taq polymerase (Invitrogen, USA), 1× PCR buffer, 1.5 mM MgCl_2_, and 2.0 mM deoxynucleotide triphosphate mix (dNTP Set; Vivantis, Malaysia). The PCR conditions were 94°C 4 min, and 30 cycles of 94°C 30 s, 55°C 30 s, and 72°C 30 s, to finalize at 72°C 3 min, carried out in a 2720 Thermal Cycler (Applied Biosystems, USA). The resulting fragment (118 bp in length) was purified by means of Centrisep columns and then sequenced using the Big Dye Terminator Kit v1.1 on ABI-310 DNA Sequencer (Applied Biosystems). The rs2934971 and rs1058808 genotypes were obtained by real-time PCR using Custom TaqMan® SNP Genotyping Assays on an ABI-PRISM 7000 Sequence Detection System, both supplied by Applied Biosystems following the recommendations of supplier.

### Statistical analysis

The genotype and allele frequencies of each SNP, as well as the Hardy-Weinberg equilibrium (HWE) were estimated. The chi-squared test was used to compare the distribution of genotypes using Arlequin v. 3.01 software (http://cmpg.unibe.ch/software/arlequin35/). The genotypic and allelic frequencies of the five polymorphisms observed in the GMP were compared with those reported for six world populations in the “1000 Genomes Project 1KGP” including a population with Mexican ancestry (MXL) (13). Odds ratios (ORs) were calculated using the Cochran-Armitage test under classical inheritance patterns, and each polymorphism was tested with the models dominant, recessive, and codominant (14). Individual results are reported as the OR and 95% confidence interval (CI). P<0.05 was considered statistically significant. We also performed a multinomial logistic regression test in SPSSv22 software (IBM, USA) to evaluate each genotype in the three groups simultaneously.

Haplotypes were established with the five SNPs according to their location in the gene from the 5′ to the 3′ position (rs2643194 C>T, rs2517951 C>T, rs2643195 A>G, rs2934971 G>T, and rs1058808 C>G). Linkage disequilibrium (LD) analysis was performed with the haplotypes data. D' values between 0 and 0.5 were considered a low level of linkage, between 0.5 and 0.75 as moderate, and greater than 0.75 as high LD. r^2^ values >0.33 were considered acceptable for correlation between sites, along with a P value of <0.05. The Arlequin v. 3.01 software was used.

## Results

Demographic data of the three groups are reported in Table 1. Significant differences were observed for age between the GC group and the GID group (P=0.01). Also, there was a greater number of men in the GC group (1.7 to 1 proportion). On the contrary, in the GID group there was a greater number of women (1.5 to 1 proportion) (P=0.003).

**Table 1:** Demographic data of the studied groups.

	GC (n=74)	GID (n=76)	GMP (n=102)	P
Age				
Mean years	59.9	54.6	32.4	P=0.01*
(SD±)	13.6	14.3	10.0	P=0.01*
Range	29−87	20−89	18−61	P=0.01*
Sex				
Female n (%)	27 (36.5%)	46 (60.5%)	44 (43.1%)	P=0.003^#^
Male n (%)	47 (63.5%)	30 (29.5%)	58 (56.9%)	P=0.003^#^
Lauren classification				
Diffuse	35 (50%)			
Intestinal	26 (37.1%)			
Mixed	6 (8.6%)			
Lymphoma	3 (4.3%)			
Tumor differentiation				
Well	4 (7.3%)			
Moderate	24 (43.6%)			
Poor	27 (49.1%)			

GC: Gastric cancer; GID: Gastric inflammatory diseases; GMP: General Mexican Population. *t-test; ^#^chi-squared test.

### Genotypic and allelic frequencies

The genotypic frequencies of all five SNPs in the three groups showed that heterozygous genotypes had the highest frequencies (range: 0.45−0.58), and that the mutated genotypes had higher frequencies than the wild-type genotypes (ranges: 0.24−0.39 *vs* 0.07−0.31, respectively). We also observed a higher frequency of mutated than wild-type alleles in terms of allelic frequencies of the five SNPs except for GID (controls) in the rs2517951, rs2643195, and rs1058808 polymorphisms (Supplementary Table S1). HWE was observed in all groups for all SNPs (P>0.05).

The distribution of allelic and genotypic frequencies for each SNP was compared between the three studied groups; significant differences were observed in allelic frequencies between GC and GID groups of the rs2643194 (P=0.03) and rs2934971 (P=0.02) polymorphisms, as well as in allelic frequencies between GID and GMP groups of the rs2517951 (P=0.03), rs2643195 (P=0.03), rs2934971 (P=0.01), and rs1058808 (P=0.01), and in the genotypic frequencies for rs2934971 (P=0.005) and rs1058808 (P=0.01) (Supplementary Table S1).

### Risk analysis

The Cochran-Armitage test showed that three SNPs were significantly associated with increased risk for GC under a dominant inheritance model: rs2643194 (CT+TT *vs* CC; OR=2.75, 95%CI 1.12−6.75, P=0.023); rs2934971 (GT+TT *vs* GG, OR=2.41, 95%CI 1.01−5.76, P=0.043); rs1058808 (CG+GG vs CC, OR=2.21, 95%CI 1.00−4.87, P=0.046). rs2517951 and rs2643195 were not associated with risk of GC. The analysis of multinomial logistic regression showed results that are in accordance with those observed in the Cochran-Armitage test.

### Haplotypes

Haplotype analysis showed 11 of the 32 possible combinations in the three studied groups (Table 2). The GC and GMP groups had nine haplotypes each, while the GID group had five. The two most frequent haplotypes in the three groups were TTGTG (>46.7%) and CCAGC (>34.7%), while the remaining combinations were observed at frequencies less than 5%. A comparison of the distribution of haplotypes between the GMP *vs* the GID and GC groups did not show any significant differences (Table 2); however, the wild haplotype “CCAGC” was observed in a higher proportion in the GID group (P=0.02); the risk analysis for this haplotype showed an OR of 0.57 (95%CI 0.36-0.92, P=0.02).

**Table 2:** Haplotypes observed in the studied groups.

Haplotype	Sites					GC	GID	GMP
	I	II	III	IV	V	n=144	n=150	n=196
	C>T	C>T	A>G	G>T	C>G	(%)	(%)	(%)
1	T	T	G	T	G	78 (54.1)	70 (46.7)	112 (57.2)
2	C	C	A	G	C	50 (34.7)	72 (48.0)	72 (36.8)
3	T	C	G	T	C	3 (2.1)	4 (2.7)	4 (2.0)
4	T	C	A	T	C	6 (4.2)	3 (2.0)	3 (1.5)
5	T	C	A	T	G	2 (1.4)		1 (0.5)
6	T	T	G	T	C	1 (0.7)		1 (0.5)
7	T	C	G	T	G	1 (0.7)		1 (0.5)
8	C	T	G	T	G			1 (0.5)
9	C	C	A	G	G			1 (0.5)
10	T	C	A	G	C	2 (1.4)	1 (0.7)	
11	T	T	G	G	G	1 (0.7)		
	P value					0.12^a^0.36^b^	0.13^c^	

I: rs252643194; II: rs2517951; III: rs2643195; IV: rs2934971; V: rs1058808. GC: gastric cancer; GID: gastric inflammatory diseases; GMP: general Mexican population; n: total of chromosomes. ^a^Chi-squared test between GC *vs* GID. ^b^Chi-squared test between GC *vs* GMP. ^c^Chi-squared test between GID *vs* GMP.

### Linkage disequilibrium (LD)

In the linkage analysis of the five sites, rs2643194, rs2517951, rs2643195, rs2934971, and rs1058808, the results showed statistically significant LD for the 10 pairs of loci of each of the three groups studied (D'>0.9415, r^2^>0.6649, and P<0.0001).

### Comparison of frequencies in the general Mexican population with other populations

In general, the distribution of genotypic and allelic frequencies for all five polymorphisms in GMP was different than that in the East Asian population (P>0.05) while it was similar to the American population (P>0.05) (Table 3).

**Table 3:** Frequencies of the five single nucleotide polymorphisms reported in several populations worldwide.

	Population						
	GMP	MXL	African	American	East Asian	European	South Asian
	(n=102)	(n=67)	(n=661)	(n=347)	(n=504)	(n=503)	(n=489)
rs2643194							
CC%	12.8	16.4	10.0	19.6	35.7	8.4	11.4
CT%	51.0	50.8	45.4	47.0	47.4	45.1	37.8
TT%	36.2	32.8	44.6	33.4	16.9	46.5	50.8
		P=0.77	P=0.266	P=0.292	**P<0.001**	P=0.103	**P=0.022**
C%	38.2	42.2	32.7	43.1	59.4	30.9	30.4
T%	61.8	57.8	67.3	56.9	40.6	69.1	69.6
		P=0.51	P=0.126	P=0.229	**P<0.001**	**P=0.048**	**P=0.032**
rs2517951							
CC%	15.7	22.4	66.1	28.2	36.7	9.3	17.2
CT%	52.9	47.8	30.1	46.7	46.6	46.6	42.5
TT%	31.4	29.8	3.8	25.1	16.7	44.1	40.3
		P=0.54	**P<0.001**	**P=0.031**	**P<0.001**	**P=0.026**	P=0.143
C%	42.2	46.9	81.2	51.6	60.0	32.6	38.4
T%	57.8	53.1	18.8	48.4	40.0	67.4	61.6
		P=0.46	**P<0.001**	**P=0.021**	**P<0.001**	**P=0.012**	P=0.348
rs2643195							
AA%	12.8	17.9	43.3	21.9	35.7	8.6	11.5
AG%	53.9	49.3	42.8	47.3	47.4	44.9	37.8
GG%	33.3	32.8	13.9	30.8	16.9	46.5	50.7
		P=0.64	**P<0.001**	P=0.115	**P<0.001**	**P=0.035**	**P<0.005**
A%	39.7	43.0	64.7	45.5	59.4	31.0	30.4
G%	60.3	57.0	35.3	54.5	40.6	69.0	69.6
		P=0.60	**P<0.001**	P=0.152	**P<0.001**	**P=0.017**	**P=0.009**
rs2643195							
AA%	12.8	17.9	43.3	21.9	35.7	8.6	11.5
AG%	53.9	49.3	42.8	47.3	47.4	44.9	37.8
GG%	33.3	32.8	13.9	30.8	16.9	46.5	50.7
		P=0.64	**P<0.001**	P=0.115	**P<0.001**	**P=0.035**	**P<0.005**
A%	39.7	43.0	64.7	45.5	59.4	31.0	30.4
G%	60.3	57.0	35.3	54.5	40.6	69.0	69.6
		P=0.60	**P<0.001**	P=0.152	**P<0.001**	**P=0.017**	**P=0.009**
rs2934971							
GG%	11.1	17.9	11.5	19.9	35.7	8.6	11.0
GT%	53.5	49.3	46.1	46.7	47.4	44.9	38.2
TT%	35.4	32.8	42.4	33.4	16.9	46.5	50.8
		P=0.12	P=0.372	P=0.125	**P<0.001**	P=0.105	**P=0.012**
G%	37.9	43.0	34.6	43.2	59.4	31.0	30.2
T%	62.1	57.0	65.4	56.8	40.6	69.0	69.8
		P=0.18	P=0.384	P=0.193	**P<0.001**	P=0.068	**P=0.035**
rs1058808							
CC%	12.8	22.4	65.5	29.1	36.5	9.8	17.8
CG%	54.9	46.3	30.6	46.1	46.6	45.9	42.5
GG%	32.3	31.3	3.9	24.8	16.9	44.3	39.7
		P=0.24	**P<0.001**	**P=0.002**	**P<0.001**	P=0.077	P=0.077
C%	40.2	46.1	80.8	52.2	59.8	32.7	39.1
G%	59.8	53.9	19.2	47.8	40.2	67.3	60.9
		P=0.33	**P<0.001**	**P=0.004**	**P<0.001**	**P=0.045**	P=0.817

Data are reported as frequencies. P values are between comparisons in the frequency of GMP (General Mexican population) and each reported population. Statistically significant P values are shown in bold. MXL: population of Mexican ancestry.

## Discussion

*ERBB2* is a gene frequently altered in different types of cancer. In gastric cancer, the alterations reported included mutations (n=34 different), and amplification and overexpression (up to 22.1% and 53.4% of cases, respectively); the latter are associated with a poor survival of the patients (15). In this work, we present the results of the association analysis of five *ERBB2* SNPs with the risk of gastric cancer.

rs2643194 is a 5′ UTR variant. Mutated (TT) or heterozygote (CT) genotypes showed increased risk for gastric cancer compared to wild genotype (CC), with an OR of 2.75 (P=0.023). Similar results have been reported by other authors, such as an increased risk for lung cancer for TT+CT *vs* CC genotypes with an OR of 2.68 (95%CI 1.51−4.75, P=0.0007) in Indian patients (16), as well as in females, non-drinkers and non-smokers lung cancer Korean patients (10). Also, this SNP was associated with risk of prostate cancer in a Chinese population (17).

rs2934971, also known as –1985 G>T, showed association with GC under a dominant model, since the mutated (TT) and heterozygote GT genotypes showed increased risk for GC (OR 2.41, P=0.043); however, this SNP was not related to breast or lung cancer in Korean patients (7,10).

rs1058808 (c.3418 C>G), also known as P1170A, is a missense variation located on exon 31 of the *ERBB2* gene, which results in a proline to alanine substitution in a C-terminal intracellular regulatory domain. This SNP showed an increased risk for gastric cancer with the mutated genotype (GG) or heterozygote (CG) genotype (OR 2.21, P=0.046). Similarly, this SNP was previously associated with an increased risk for rectal cancer in TP53 positive tumors in American patients (OR 1.7, P=0.03) (18). As known, HER2 receptor lacks a ligand binding domain, therefore it requires forming heterodimers with other receptors such as EGFR, HER3, or HER4 to carry out its function in the signaling pathways, such as mitogen-activated protein kinase (MAPK) and phosphatidylinositol-3 kinase (PI-3K) (4). *In silico* analysis with PolyPhen-2 software (http://genetics.bwh.harvard.edu/pph2/) showed a score of 0.953, indicating that P1170A is a damaging variant, which can impact the structure and function of HER-2 protein. Thus, this polymorphism located on a region that encodes for the intracellular regulatory domain could probably modify the signaling pathways, and consequently be involved in gastric carcinogenesis. Although our results suggested that these three polymorphisms could have a role in gastric carcinogenesis, additional studies are required in order to establish if they could modulate gene expression and, consequently, result in an imbalance between cell proliferation and apoptosis.

The SNPs rs2517951 and rs2643195 were not associated with GC in our population. However, some authors have observed an association of the rs2643195 SNP with protection against prostate cancer in Chinese patients (17), as well the rs2517951 SNP with an increased risk of endometrial cancer in Korean women with obesity (9).

Regarding the high LD observed in our study for the five SNPs(D'>0.941 and r^2^>0.665), other authors have also reported high LD within this genomic region, which covers 33.9 kb, spanning the entire coding region of the *ERBB2* gene, with D' values >0.7 (5), >0.88 (7), and >0.89 (8). Neither haplotype was associated with a significant risk for GC; no author has found an association of these haplotypes with breast cancer (5,7,8).

The similarities in the frequencies for the rs2643194, rs2517951, rs2643195, rs2934971, and rs1058808 SNPs observed among the GMP and European, American, South Asian, and African populations (Table 3) are due to the high variability in the genetic component of the Mexican Mestizo population, which is an admixture of Spanish, Native American, and African genes (19).

Finally, the SNPs rs2643194, rs2934971, and rs1058808 of the *ERBB2* gene were associated with increased risk for GC in the Mexican population. It would be interesting to deepen the study of these markers and their role in such diseases in other populations, as well as to know the levels of expression of the HER2 receptor in this group of alterations.

## Supplementary Material

Click here to view [pdf].
